# Machine-learning algorithm-based prediction of a diagnostic model based on oxidative stress-related genes involved in immune infiltration in diabetic nephropathy patients

**DOI:** 10.3389/fimmu.2023.1202298

**Published:** 2023-07-24

**Authors:** Heng-Mei Zhu, Na Liu, Dong-Xuan Sun, Liang Luo

**Affiliations:** ^1^ Department of Nephrology, South China Hospital, Medical School, Shenzhen University, Shenzhen, Guangdong, China; ^2^ Department of Nephrology, The First Affiliated Hospital of Nanchang University, Nanchang, Jiangxi, China; ^3^ Department of Nephrology, Huazhong University of Science and Technology Union Shenzhen Hospital, Shenzhen, Guangdong, China; ^4^ Department of Nephrology, the Third Hospital of Nanchang, Nanchang, Jiangxi, China; ^5^ Department of Cardiology, Ganzhou People’s Hospital, Ganzhou, China

**Keywords:** infiltrating immune cells, diagnostic, biomarker, diabetic nephropathy, machine learning, oxidative stress-related genes

## Abstract

Diabetic nephropathy (DN) is the most prevalent microvascular consequence of diabetes and has recently risen to the position of the world’s second biggest cause of end-stage renal diseases. Growing studies suggest that oxidative stress (OS) responses are connected to the advancement of DN. This study aimed to developed a novel diagnostic model based on OS-related genes. The differentially expressed oxidative stress-related genes (DE-OSRGs) experiments required two human gene expression datasets, which were given by the GEO database (GSE30528 and GSE96804, respectively). The potential diagnostic genes were identified using the SVM-RFE assays and the LASSO regression model. CIBERSORT was used to determine the compositional patterns of the 22 different kinds of immune cell fraction seen in DN. These estimates were based on the combined cohorts. DN serum samples and normal samples were both subjected to RT-PCR in order to investigate the degree to which certain genes were expressed. In this study, we were able to locate 774 DE-OSRGs in DN. The three marker genes (DUSP1, PRDX6 and S100A8) were discovered via machine learning on two different machines. The high diagnostic value was validated by ROC tests, which focused on distinguishing DN samples from normal samples. The results of the CIBERSORT study suggested that DUSP1, PRDX6, and S100A8 may be associated to the alterations that occur in the immunological microenvironment of DN patients. Besides, the results of RT-PCR indicated that the expression of DUSP1, PRDX6, and S100A8 was much lower in DN serum samples compared normal serum samples. The diagnostic value of the proposed model was likewise verified in our cohort, with an area under the curve of 9.946. Overall, DUSP1, PRDX6, and S100A8 were identified to be the three diagnostic characteristic genes of DN. It’s possible that combining these genes will be effective in diagnosing DN and determining the extent of immune cell infiltration.

## Introduction

Diabetic nephropathy (DN) is a major complication of type I and II diabetes and the main cause of end stage renal disease (1). The clinical and pathological features of DN are characterized by a progressive increase in albuminuria, a decline in glomerular filtration rate and the loss of podocytes (2, 3). Morphologically speaking, DN is distinguished by alterations in the thickness and composition of the glomerular basement membrane, in addition to mesangial enlargement, which ultimately results in tubulointerstitial fibrosis (4, 5). Diabetes can harm various organs throughout the body, such as the kidneys, eyes, heart and more. In addition, in diabetic nephropathy, inflammatory responses and the release of inflammatory mediators play a crucial role. These inflammatory mediators can lead to vascular changes, fibrosis, and tissue damage, affecting the structure and function of peripheral nerves (6). Persistent hyperglycemia and long-term metabolic abnormalities also contribute to this damage (7, 8). Diabetes and its complications not only affect the physical and mental health of individuals but also have a significant impact on society, both economically and socially (9). This is because the prevalence of diabetes is increasing across the globe for people of all ages. In China, the number of people suffering from diabetic nephropathy has been steadily rising over the past several years. At the moment, the primary methods of treatment for diabetic nephropathy in both the United States and other countries include decreasing blood pressure, controlling hypoglycemia, and managing lipid levels (10, 11). Although these treatments can slow the progression of the disease, there is currently no cure or particular medication that can reverse or eliminate it. As a result, it is of the utmost need to do more research into its pathophysiology in order to locate reliable biomarkers for the early identification and treatment of the condition.

Oxidative stress (OS) reactions are reported to be associated with the progression of DN (12, 13). High blood glucose levels contribute to increased oxidative stress through spontaneous glucose oxidation and the formation of advanced glycation end products. Additionally, mitochondrial dysfunction, inflammation, and activation of the renin-angiotensin system also contribute to ROS generation. OS triggers extracellular matrix accumulation, endothelial cell dysfunction, podocyte injury, and inflammation in DN, further exacerbating renal damage (14, 15). It is possible that the pathogenetic processes that cause macro- and micro-vascular problems are the same, with reactive oxygen species (ROS) serving as the common denominator of diverse signaling pathways that ultimately result in an attack on multiple target organ systems (16, 17). The ROS are a family of molecules including molecular oxygen and its derivatives, nitric oxide (NO), hypochlorous acid (HOCl), peroxynitrite (ONOO−), hydrogen peroxide (H2O2), hydroxyl racial (HO·), superoxide anion (O2−) and lipid radicals (18, 19). Many ROS have electrons that are unpaired, and as a result, they are considered to be free radicals. After being able to overcome the numerous endogenous anti-oxidative defense systems, excessive concentrations of ROS will oxidize various tissue biomolecules, including as DNA, protein, carbohydrates, and lipids. This perilous state is usually referred to as an oxidative stress (20, 21). Imaging tests, molecular diagnostics, and histological examinations are the primary pillars around which DN diagnostic procedures are built. For instance, MRI (Magnetic Resonance Imaging) utilizes a powerful magnetic field and radio waves to generate detailed images of the kidneys. It can provide information about kidney structure, blood flow, and any signs of damage or disease (22). Molecular diagnostic tests can measure the levels of specific biomarkers in blood or urine, which indicate the presence of DN. For example, measuring urine protein, urine albumin, or specific cytokine levels can help assess kidney damage and the severity of DN (23). Renal biopsy involves obtaining small tissue samples from the kidney for microscopic examination. It can evaluate histological changes in the kidney, such as glomerular basement membrane thickening, mesangial expansion, and tubulointerstitial fibrosis. This examination provides valuable information about the extent and severity of kidney damage in DN (24). There is just a limited subset of OS-related genes(TXNIP and NLRP3) that have been subjected to in-depth research and are known to play an important part in the development of DN (25–27). Gene expression data have recently been supplied by large-scale genome profiles, which gives a good opportunity to find relevant molecular markers. In addition, bioinformatic study of OS genes may assist in the identification of new diagnostic or prognostic indicators for DN, which may then be used to search for novel therapy targets (28, 29). In addition, studies have demonstrated that immune cell infiltration plays an increasingly critical role in the onset and progression of a wide variety of illnesses, including DN, which provides unique insights into the relevance of immune regulation in DN (30, 31). For instance, monocyte infiltration and subsequent differentiation into macrophages have been observed in the kidneys of individuals with DN. These macrophages contribute to inflammation and fibrosis in the renal tissue (32). Dendritic cells have been found in the kidneys of individuals with DN. They play a role in antigen presentation and the activation of immune responses in the renal tissue (33).

For the purpose of this investigation, we accessed the GEO database and downloaded two microarray datasets. Analyses of differentially expressed oxidative stress-related genes (DE-OSRGs) were carried out on the DN and the controls. Algorithms based on machine learning were utilized in order to screen for and locate diagnostic biomarkers of DN. The diagnostic prediction model was constructed with the use of a logistic regression technique, and candidate genes that are highly connected to immune infiltration were found, verified, and employed in the process. Our findings provided a novel model that may be used to forecast the diagnosis of DN patients, and it gave a fresh viewpoint for DN therapy targets. In addition, our findings presented a novel model that might be used to diagnose DN patients.

## Materials and methods

### Serum samples

25 DN patients (14 males and 11 females, aged between 40 and 70 years old) and 25 healthy donors(13 males and 12 females, aged between 43 and 68 years old) in South China Hospital were involved in this study. The patients had not received any therapy for DN before sample collection. All participants provided written informed consent. This study was approved by the Ethics Committee of South China Hospital. After collecting blood samples from patients with DN and healthy donors, centrifuges were used to separate serum samples from the blood. Before they are utilized, serum samples are kept cold in the refrigerator at a temperature of 80 degrees Celsius.

### RNA extraction and real-time PCR

The TRIzol reagent was used in order to extract the total RNA from the sample. Following the protocol provided by the manufacturer, one microgram of total RNA was used to produce one nanogram of first-strand complementary DNA (cDNA) using the Reverse Transcription System Bestar qPCR RT Kit. An ABI 7500 Real-Time PCR System was utilized for the execution of the real-time PCR (Applied Biosystems, China). Every experiment was carried out with three separate replicates, and the beta-actin gene served as the internal control. The relative expressions of Dual Specificity Phosphatase 1(DUSP1), Peroxiredoxin 6(PRDX6), and S100 calcium-binding protein A8(S100A8) were calculated using with a 2^−ΔΔCt^ method and normalized using GAPDH as an internal control. The primers used in this study were shown below: for DUSP1, 5’-AGTACCCCACTCTACGATCAGG-3’ (forward), 5’- GAAGCGTGATACGCACTGC-3’(reverse); for PRDX6, 5’- GACTCATGGGGCATTCTCTTC-3’ (forward), 5’- CAAGCTCCCGATTCCTATCATC-3’(reverse); S100A8, 5’- ATGCCGTCTACAGGGATGAC-3’ (forward), 5’- ACTGAGGACACTCGGTCTCT-3’(reverse); GAPDH, 5’-CTGGGCTACACTGAGCACC-3’ (forward), 5’- AAGTGGTCGTTGAGGGCAATG-3’(reverse).

### Microarray data and preprocessing

“diabetic kidney disease,” “diabetic nephropathy,” and “expression profiling by array” were the search phrases that were used in order to retrieve the mRNA expression data as well as the associated experimental and clinical data of DN from GEO. It was decided to choose and download the gene expression microarray datasets GSE30528 and GSE96804 respectively. The relative expressions of all genes from the above two datasets were calculated using with a 2^−ΔΔCt^ method. The OSRGs, totaling 1,399, were retrieved from the GSEA website that is accessible online.

### Identification of differentially expressed genes

We began by pulling expression data for 1399 OSRGs from the GSE96804 database. These samples included normal samples as well as DN samples. After that, the student’s t-test was carried out in R in order to identify the OSRGs that had distinct levels of expression within the two distinct samples. Genes that had a p-value of less than 0.05 were regarded to be significant.

### Functional pathway analyses

GO and KEGG analyses were performed using the clusterProfiler (version 3.10.1) package in order to uncover the possible gene functional annotation and pathway enrichment related with the common DE-OSRGs. This was done so that the prospective gene functional annotation could be shown. The enrichplot and DOSE packages were utilized in order to provide visualization of the enrichment results, which assisted in interpretation. P value less than 0.05 and adjusted P value less than 0.05 were chosen as the cutoff criteria. In addition, Disease ontology (DO) enrichment analyses were carried out on DE-OSRGs with the help of the “clusterProfiler” and DOSE packages in the R programming language.

### Identification of optimal diagnostic gene biomarkers for DN patients

In order to lessen the amount of space used by the data, LASSO method was used in conjunction with the glmnet package. The DE-OSRGs that differentiated DN patients from normal samples were kept for feature selection, and the LASSO algorithms were used to locate gene biomarkers that were associated with DN. During this time, a SVM-RFE model was developed using an SVM software. This model and others were evaluated based on the average misjudgement rates of their 10-fold cross-validations. In addition, the overlapping biomarkers that were obtained from the two algorithms were used to locate the most effective gene biomarkers for DN. In addition, the predict function found within the glm package of the R programming language was utilized to build a logistic regression model that was based on three marker genes. This model was then used to make predictions regarding the sample types found within the GSE30528 and GSE96804 datasets. In a similar manner, ROC curves were utilized in order to assess the diagnostic capability of the logistic regression model.

### Immune infiltration analysis

CIBERSORT is the most widely mentioned tool for estimating and assessing the number of immune cells that have infiltrated a given area. It is a method that characterizes the cell composition based on the gene expression patterns of the cells. We were able to determine the proportions of immune cell types that were present in low-expression and high-expression groups by using CIBERSORT. Each sample has a total score of one, which corresponds to the sum of all the projected values for the different immune cell types.

### Statistical analysis

R(Version 3.5.0) was utilized throughout all of the statistical work that was completed. The Student’s t-test and the chi-square test were used to investigate and compare the results of the various groups. Statistical significance was assigned to the p-values when they were lower than 0.05.

## Results

### Identification of DE-OSRGs in DN patients

**Figure 1:**
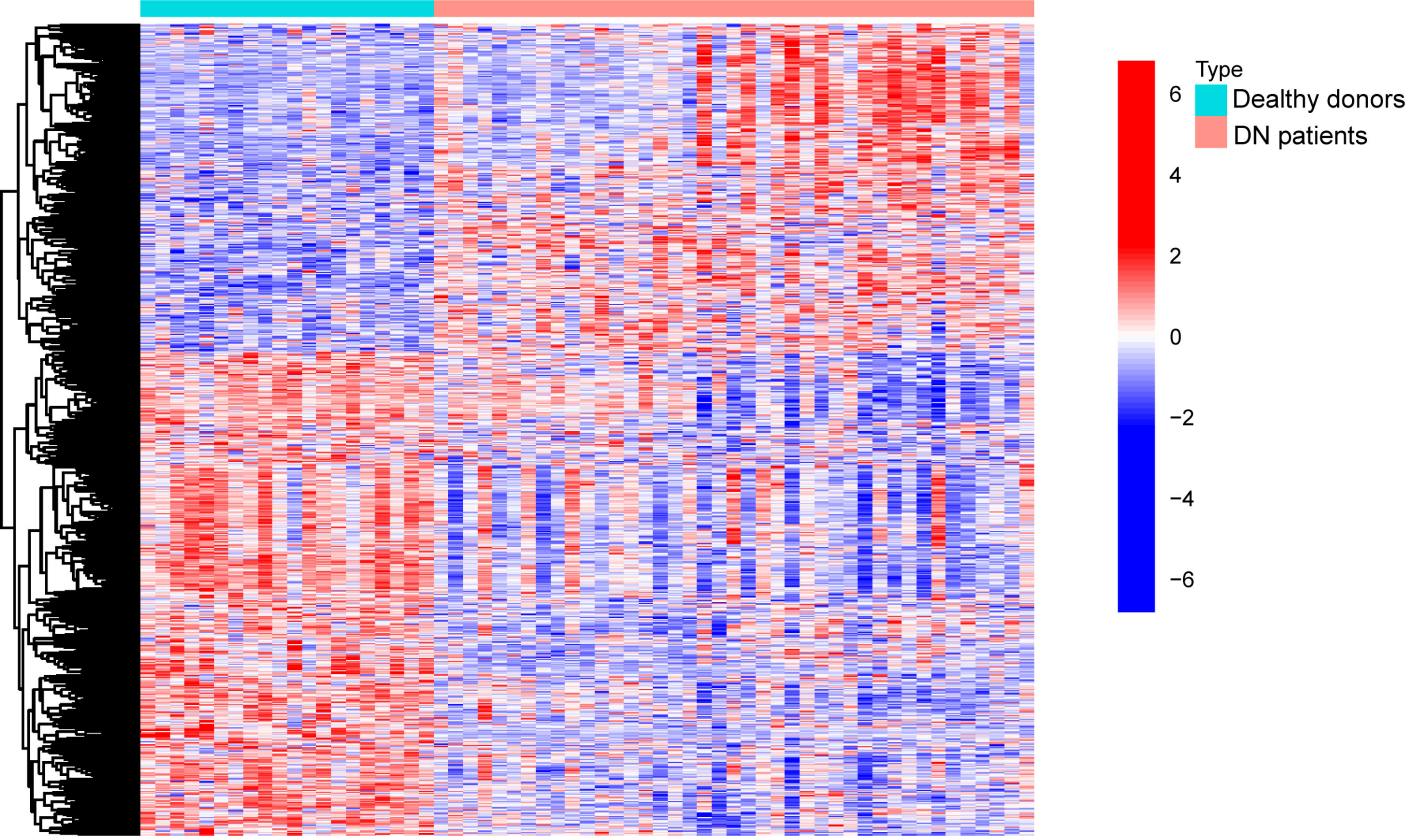
Comparison of DN samples with normal samples for the purpose of DE-OSRG identification.

In this work, a retrospective analysis was performed on the data from 41 DN samples and 21 normal samples from GSE96804. The study yielded 774 differentially expressed genes (DEGs), with 312 genes showing significant upregulation and 462 genes showing significant downregulation (**Figure 1**).

### Functional enrichment analysis of DE-OSRGs

**Figure 2:**
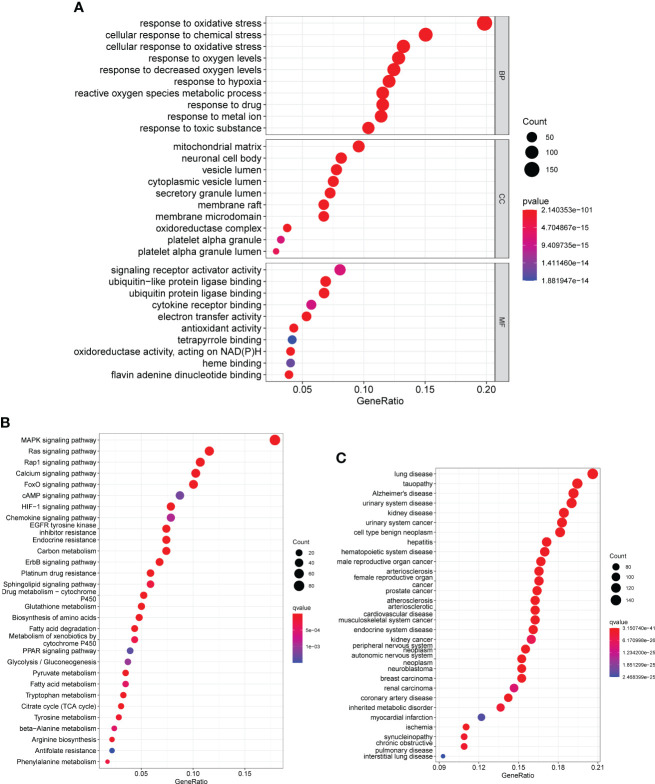
GO, KEGG and DO enrichment analysis of DE-OSRGs. **(A)** BP, CC, and MF are among the most highly enriched GO keywords of downregulated and upregulated DE-OSRGs, respectively. **(B)** KEGG pathway enrichment analysis of DE-OSRGs. **(C)** Disease ontology enrichment analysis of DE-OSRGs.

After that, we used the “clusterProfiler” tool to perform gene ontology (GO) analysis on the DE-OSRGs. This analysis helped us to understand the biological functions and pathways associated with these DE-OSRGs, providing insights into the underlying mechanism of the disease. As shown in **Figure 2A**, the DE-OSRGs were mainly involved in response to cellular response to chemical stress, mitochondrial matrix, cellular response to oxidative stress, oxidative stress, neuronal cell body, vesicle lumen, signaling receptor activator activity, ubiquitin-like protein ligase binding and ubiquitin protein ligase binding. Moreover, we performed KEGG analysis and observed that the DE-OSRGs were mainly involved in MAPK signaling pathway, Rap1 signaling pathway, Ras signaling pathway, Calcium signaling pathway, FoxO signaling pathway, HIF-1 signaling pathway and cAMP signaling pathway (**Figure 2B**). In addition, the results of DO analysis indicated that the DE-OSRGs were associated with lung disease, tauopathy, Alzheimer’s disease, urinary system disease, kidney disease and urinary system cancer (**Figure 2C**).

### Three DE-OSRGs were identified as diagnostic genes for DN

**Figure 3:**
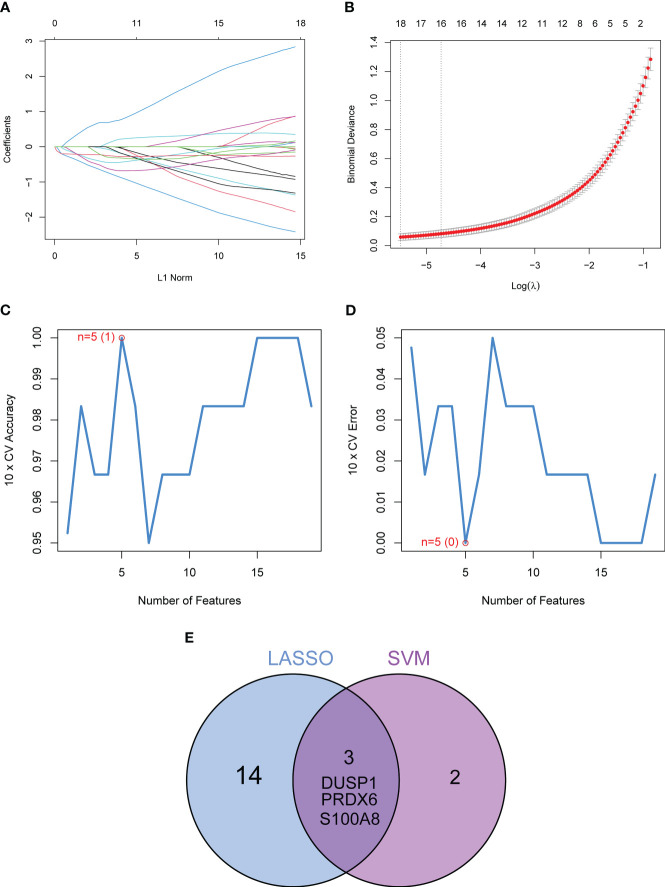
Three DE-FGs were identified as diagnostic genes for DN. **(A, B)** In order to pick 18 DN-related features, the LASSO logistic regression technique was utilized, and the penalty parameter tuning was carried out using 10-fold cross-validation. **(C, D)** The SVM-RFE technique was used to filter the DE-FRGs in order to determine the best combination of feature genes. **(E)** We obtained a set of marker genes from the LASSO and SVM-RFE models.

**Figure 4:**
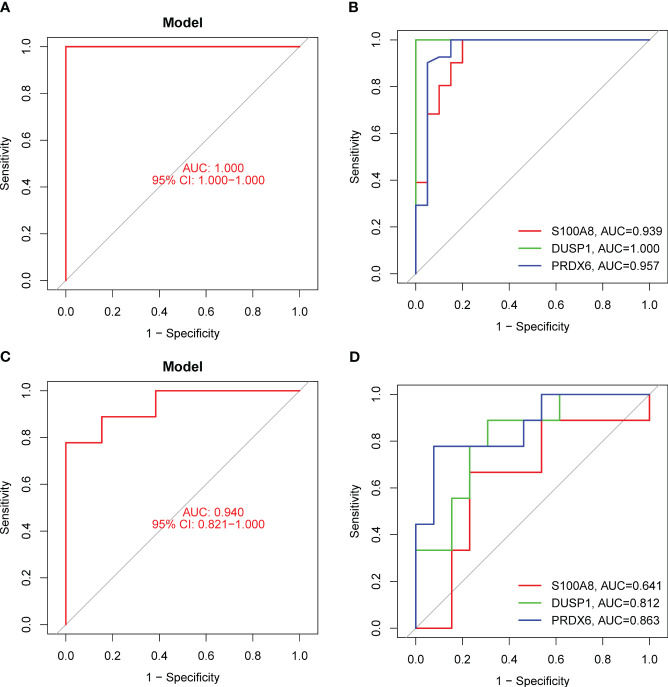
The diagnostic value of the new diagnostic model for DN. **(A)** Logistic regression model was applied for the identification of the AUC of DN samples using GSE96804 datasets. **(B)** ROC curves for DUSP1, PRDX6 and S100A8 using GSE96804 datasets. (**C** and **D**) The diagnostic value of the new model was further demonstrated in GSE30528 datasets.

In order to evaluate the potential of DE-OSRGs as diagnostic markers for DN, we applied two machine learning algorithms, LASSO and SVM-RFE, on the GSE96804 dataset. These algorithms were used to identify a set of significant DE-FRGs. In order to choose 18 DN-related features, the LASSO logistic regression technique was utilized, and the penalty parameter tuning process was carried out using 10-fold cross-validation (**Figures 3A, B**). Following this, we filtered the DE-OSRGs using the SVM-RFE method in order to locate the best possible combination of feature genes. In the end, five genes were selected as the best candidates for feature genes (**Figures 3C, D**). After intersecting the diagnostic factors generated from the LASSO and SVM-RFE methods, three marker genes (DUSP1, PRDX6, and S100A8) were selected for further investigation (**Figure 3E**). We constructed a novel diagnostic model using the R package glm based on the aforementioned three marker genes, and we observed that the model could distinguish normal and DN samples with a perfect accuracy (AUC=1.000) (**Figure 4A**). In addition, ROC curves were constructed for the three marker genes in order to shed light on the capability of individual genes to differentiate DN samples from normal samples. As shown in **Figure 4B**, The AUC was more than 0.7 for each and every gene. In addition, in order to give more evidence of the diagnostic usefulness of our model, we conducted additional research on the GSE30528 datasets. The findings revealed that the new model delivered a higher level of accuracy and specificity (**Figures 4C, D**).

### The expressing pattern of DUSP1, PRDX6 and S100A8 in DN patients

**Figure 5:**
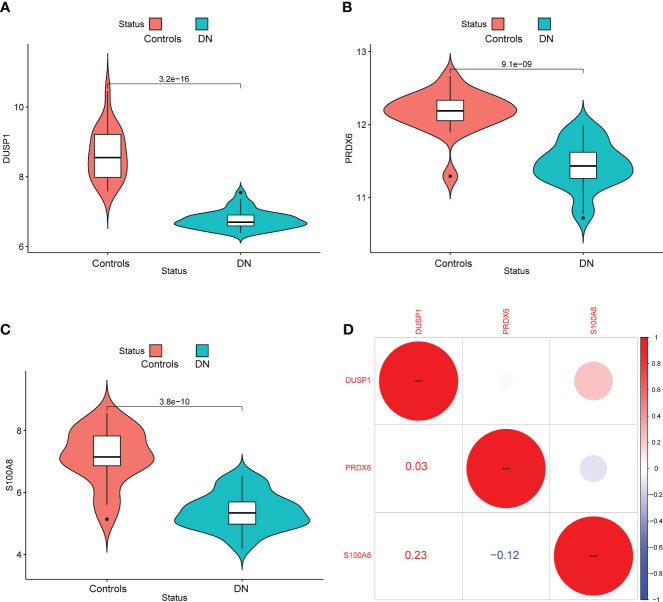
The expressing pattern of DUSP1, PRDX6 and S100A8 in GSE96804 cohorts. The expression of **(A)** DUSP1, **(B)** PRDX6 and **(C)** S100A8 was distinctly decreased in DN samples compared with normal samples. **(D)** The correlation of DUSP1, PRDX6 and S100A8.

**Figure 6:**
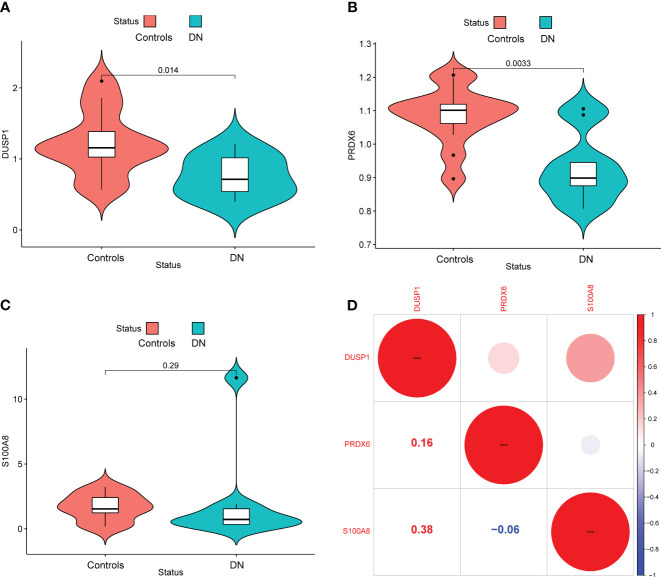
The expressing pattern of DUSP1, PRDX6 and S100A8 in GSE30528 cohorts. The expression of **(A)** DUSP1, **(B)** PRDX6 and **(C)** S100A8 was distinctly decreased in DN samples compared with normal samples. **(D)** The correlation of DUSP1, PRDX6 and S100A8.

In addition, we showed the expressing pattern of DUSP1, PRDX6, and S100A8 in DN from the GSE96804 datasets and discovered that the expressions of DUSP1, PRDX6, and S100A8 were markedly reduced in DN samples in comparison with normal samples. This finding was based on the fact that the expression of these genes was significantly increased in normal samples (**Figures 5A–C**). The correlation between these genes was presented in **Figure 5D**. The level of DUSP1 expression was shown to have a positive correlation with the level of S100A8 expression. In addition, we found that the expressions of DUSP1 and PRDX6 were distinctly lower in samples of patients with DN as compared to those of patients with normal blood pressure (**Figures 6A–C**). The correlation between these genes was presented in **Figure 6D**. The level of DUSP1 expression was shown to have a positive correlation with the levels of PRDX6 and S100A8 expression.

### Relationship between DUSP1, PRDX6 and S100A8 with the proportion of infiltrating immune cells

**Figure 7:**
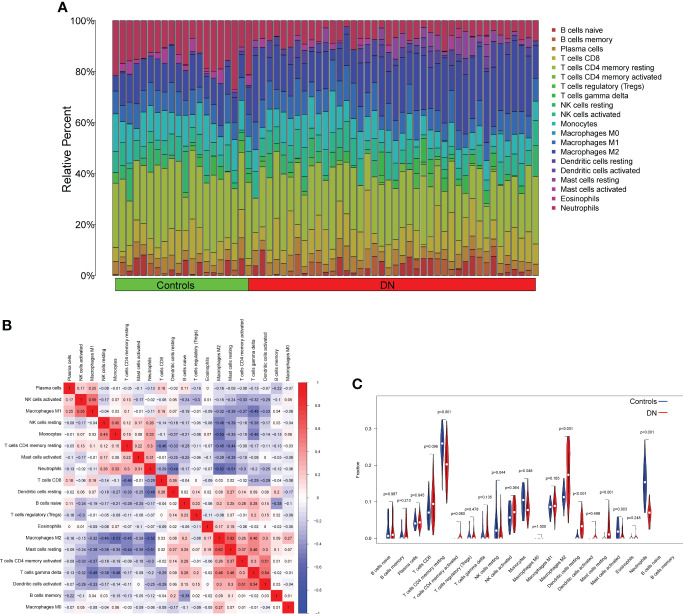
Infiltrating immune cells profiles in DN samples and correlation assays. **(A)** A bar plot comparing the percentage of 21 different types of immune cells found infiltrating DN samples with those seen in normal samples. **(B)** Heatmap showing the correlation between 21 kinds of Infiltrating immune cells. **(C)** V DN samples and normal samples were compared using an iolin plot, which displayed the ratio differentiation of 21 distinct types of immune cells.

**Figure 8:**
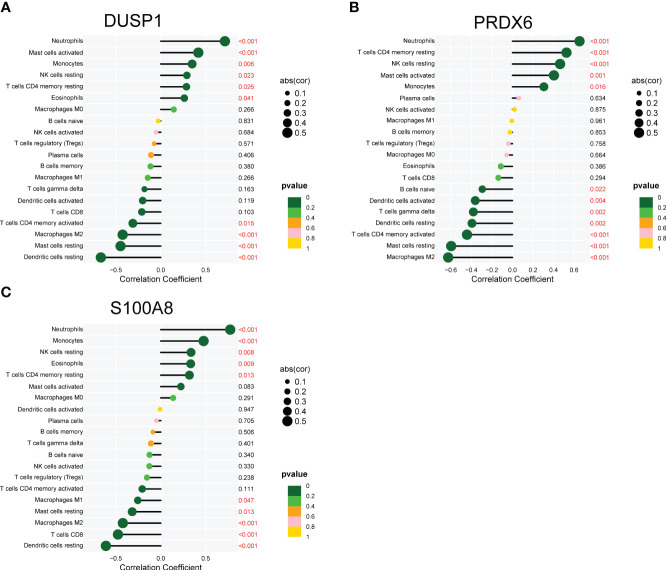
Correlation between **(A)** DUSP1, **(B)** PRDX6 and **(C)** S100A8, and infiltrating immune cells in DN.

We used the CIBERSORT approach to further validate the association between the expression of DUSP1, PRDX6, and S100A8 and the immunological component. Specifically, we constructed 21 different types of immune cell profiles in DN patients and analyzed the fraction of invading immune subtypes (**Figures 7A, B**). In addition, several immune cells exhibited a dysregulated level between DN samples and normal samples, such as T cells CD4 memory resting, NK cells resting, Monocytes, Macrophages M2, Dendritic cells resting, Mast cells resting, Mast cells activated and Neutrophils (**Figure 7C**). Then, we found that the level of DUSP1 was positively associated with Neutrophils, Mast cells activated, Monocytes, NK cells resting, T cells CD4 memory resting and Eosinophils, while negatively associated with Dendritic cells resting, Mast cells resting, Macrophages M2 and T cells CD4 memory activated (**Figure 8A**). The level of PRDX6 was positively associated with Neutrophils, T cells CD4 memory resting, NK cells resting, Mast cells activated and Monocytes, while negatively associated with Macrophages M2, Mast cells res, T cells CD4 memory activated, Dendritic cells resting, T cells gamma delta and Dendritic cells activated (**Figure 8B**). The level of S100A8 was positively associated with Neutrophils, Monocytes, NK cells resting, Eosinophils and T cells CD4 memory resting, while negatively associated with Dendritic cells resting, T cells CD8, Macrophages M2, Mast cells resting and Macrophages M1 (**Figure 8C**).

### Identification of the expression and diagnostic value of DUSP1, PRDX6 and S100A8 in our cohort

**Figure 9:**
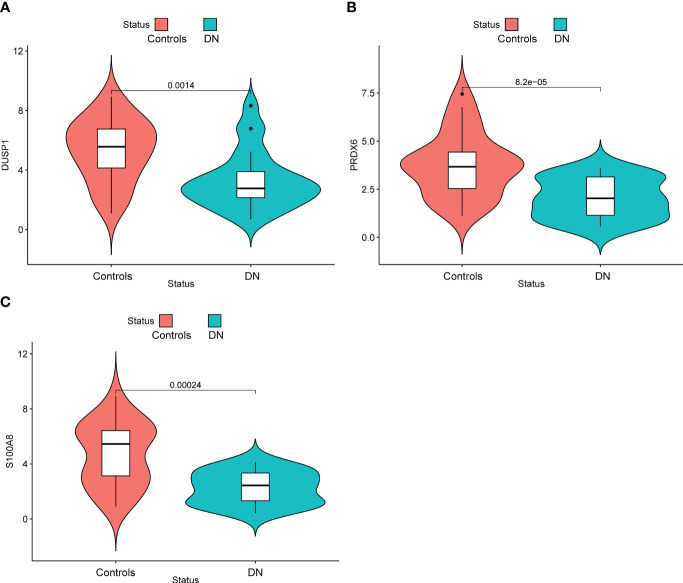
Identification of the expression of **(A)** DUSP1, **(B)** PRDX6 and **(C)** S100A8 in our cohorts.

**Figure 10:**
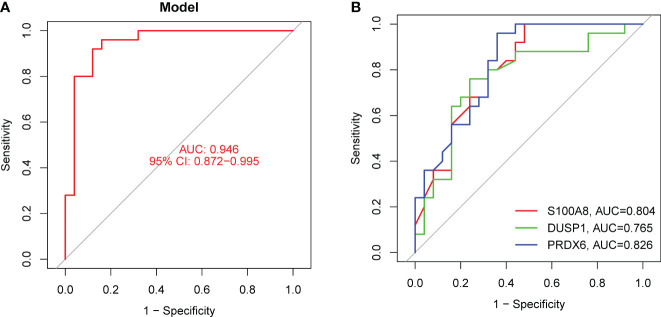
The diagnostic value of the new diagnostic model in our cohort. **(A)** The new model showed a strong ability in screening DN samples from normal samples. **(B)** ROC assays for DUSP1, PRDX6 and S100A8.

In order to provide further evidence of the aforementioned findings, we obtained 25 serum samples from patients diagnosed with DN and 25 serum samples from normal participants. According to the findings of RT-PCR, the expression of DUSP1, PRDX6, and S100A8 was much lower in the serum samples of DN patients than it was in the serum samples of normal participants (**Figures 9A–C**). Following ROC curve analysis revealed that the new model distinguished normal samples from DN samples with an AUC value of 9.946 (**Figure 10A**). In addition, ROC curves were constructed for the three marker genes in order to shed light on the capability of individual genes in discriminating DN samples from normal samples. The AUC was higher than 0.7 for every gene, as shown in **Figure 10B**. Our findings were in agreement with the data presented above.

## Discussion

DN is a common chronic complication of diabetes that can lead to glomerular hypertrophy, thickening of the basement membrane, glomerulosclerosis, and renal interstitial fibrosis (34, 35). All of these conditions can eventually result in renal failure, which poses a significant risk to human life and health safety. It is required to create novel indicators and possible targets at the molecular level in order to prevent and treat diabetic nephropathy because of the poor understanding of its pathophysiology and therapy (36, 37). In addition, DN manifests itself as glomerular damage, glomerular hypertrophy, and thickening of the glomerular basement membrane. Patients who have DN typically have a poor renal prognosis since their condition is often misdiagnosed and difficult to treat (38, 39). This may be one factor that contributes to the poor renal prognosis. However, DN is the consequence of several genes interacting with one another, and the molecular processes behind DN are still poorly understood due to the intricacy of the etiologic variations. As a result, there is an immediate need for viable biomarkers that may be used for early diagnosis and focused therapy.

Damage to tubular epithelial cells, glomerular sclerosis, apoptosis, inflammatory infiltration, and renal interstitial fibrosis are some of the usual pathological hallmarks of DN (40, 41). The exact steps that lead up to the development of DN are not yet fully understood. There is a growing body of research that points to oxidative stress and inflammation as the primary contributors to the development of DN (3, 42). Local chronic inflammatory stress not only causes organ damage and cell death directly, but it also weakens the antioxidant defense mechanisms, which further exacerbates the vicious cycle described above (43, 44). When considered as a whole, the course of DN is characterized by an interaction between oxidative stress and inflammation. In this study, we identified a total of 774 DE-OSRGs in DN patients. KEGG analysis and observed that the DE-OSRGs were mainly involved in MAPK signaling pathway, Ras signaling pathway, Rap1 signaling pathway, Calcium signaling pathway, FoxO signaling pathway, cAMP signaling pathway and HIF-1 signaling pathway. In addition, the results of DO analysis indicated that the DE-OSRGs were associated with lung disease, tauopathy, Alzheimer’s disease, urinary system disease, kidney disease and urinary system cancer. According to the results of our investigation, the function of 774 DE-OSRGs appeared to be rather complicated. Then, we carried out two different machine-learning methods, and one of the results was the identification of three essential diagnostic genes, including DUSP1, PRDX6, and S100A8. DUSP1 encodes a protein belonging to the protein tyrosine phosphatase family and was initially discovered in cultured mouse cells. It is a member of the family of threonine-tyrosine dual-specificity phosphatases. According to the findings of a prior investigation, DUSP1 is able to act as a negative regulator for the MAPK signaling pathway, and it is expressed in more than one cell line. Another, more recent work revealed that DUSP1 prevented renal cell death in DN by inhibiting the process that involves JNK, Mff, and mitochondrial fission (45, 46). PRDX6 is a gene that encodes a protein belonging to the Peroxiredoxin family. PRDX6 has multiple important functions within cells. As a peroxidase, it primarily participates in the removal of intracellular peroxides and antioxidant reactions. PRDX6 catalyzes the reduction reaction of various substrates, including hydrogen peroxide (H2O2), organic peroxides, and phospholipid hydroperoxides, thus protecting cells from oxidative stress damage. Additionally, PRDX6 exhibits phospholipase A2 (Phospholipase A2) activity, catalyzing the hydrolysis of phosphatidylcholine, and participating in cell membrane phospholipid metabolism and signal transduction processes. In addition, PRDX6 plays a significant role in various physiological and pathological processes. It is involved in regulating cellular oxidative stress responses and maintaining intracellular redox balance. The loss or abnormal expression of PRDX6 is associated with the occurrence and development of several diseases (47). Research has found that PRDX6 is important in tumor development, cardiovascular diseases, neurological disorders, inflammatory conditions, and lung diseases. It can influence cell proliferation, apoptosis, migration, and invasion capabilities, regulate vascular function and myocardial protection, and participate in neurodevelopment and the occurrence of neurodegenerative diseases (48, 49). Importantly, a previous study reported that upregulation of PRDX6 expression prevented podocyte injury in DN via regulating ferroptosis and oxidative stress (50). S100A8 encodes a protein that belongs to the S100 protein family. The S100A8 protein plays a significant role in the immune system and inflammation processes. It is a calcium-binding protein that can bind to calcium ions and participate in various cellular signaling and regulatory processes. S100A8 is typically present as a dimer and forms a complex with S100A9 protein, known as calprotectin (51, 52). The expression of S100A8 is influenced by various stimuli and regulatory factors. It is highly expressed in monocytes, neutrophils, macrophages, epithelial cells, and some inflammation-related tumor cells. Under conditions such as inflammation and infection, the expression level of S100A8 is typically elevated. It can interact with multiple receptors, such as RAGE (Receptor for Advanced Glycation End Products), TLR4 (Toll-like Receptor 4), and CD36, to participate in inflammation signaling, cell migration, and immune regulation processes (53, 54). To date, the expression and function of DUSP1, PRDX6, and S100A8 in DN were rarely reported. We began by developing a diagnostic model that made use of DUSP1, PRDX6, and S100A8. This model demonstrated significant diagnostic capabilities in both the GSE30528 and the GSE96804 cohorts. In addition, we validated the diagnostic potential of the new model in our sample population. The significance of our findings was brought to light by the possible application of the new model as an innovative diagnostic biomarker for DN.

Immunological processes play an important part in the development and progression of DN (55, 56). These mechanisms involve activating innate immune cells and producing proinflammatory chemicals. These cells play a critical role in the body’s immune response by detecting and responding to pathogens, and proinflammatory chemicals are used to signal the presence of an infection and recruit other immune cells to the site (38, 57). For instance, Macrophages are important participants in the inflammatory response. They can release inflammatory mediators, promote inflammation, and contribute to kidney damage. Specific subsets of T cells play a key role in the development of DN (32). The activation of inflammatory Th1 cells is associated with glomerular inflammation, while insufficient function of regulatory T cells (Tregs) may lead to immune tolerance imbalance and increased inflammatory response (44). Dendritic cells are antigen-presenting cells that can activate and regulate T cell immune responses and play an important role in the inflammatory process of DN (33). In addition, these mechanisms also include the production of cytokines and chemokines, which are signaling molecules that are involved in cell signaling and communication, and play a critical role in the body’s immune response. The expression of several immune and inflammatory genes is increased in diabetes, both in animal models and human patients. This elevation in gene expression leads to a chronic inflammation state in the kidneys, and contributes to the development of diabetic nephropathy, a common complication of diabetes. Moreover, the activation of these genes also contributes to the systemic inflammation, which can cause damage to other organs in the body, such as the heart and blood vessels, increasing the risk of cardiovascular disease. Patients with diabetes also have this phenomenon. These genes play a significant part in both the beginning stages of inflammation as well as the process of immune cell recruitment. CIBERSORT is a deconvolution approach that analyzes the gene expression patterns of cells in complicated tissue to determine the cell composition of the tissue (58). In order to deconvolve a combination of gene expression, it uses a technique known as linear support vector regression (SVR), which is a form of machine learning. Because its findings have been proven to correlate well with flow cytometric analysis, it has also been referred to as “digital cytometry,” which is an alternative name for the technique. Although this method has been used to treat several diseases, its application in clinical practice has been somewhat restricted. In this study, we found that the level of DUSP1 was positively associated with Neutrophils, Mast cells activated, Monocytes, NK cells resting, T cells CD4 memory resting and Eosinophils, while negatively associated with Dendritic cells resting, Mast cells resting, Macrophages M2 and T cells CD4 memory activated. In addition, PRDX6 and S100A8 were also found to be associated with several immune cells. There is evidence that T cells play a role in the development of DN. Studies in animal models have found that certain types of T cells, such as CD6+ and CD4+, are moderately increased in patients with type 2 diabetes and are linked with proteinuria (59, 60). However, more research is needed to understand the exact mechanisms by which T cells contribute to the development of DN. Additionally, it’s also possible that other immune cells and molecules may play a role in the progression of DN, and further studies are necessary to fully understand the complex interplay between the immune system and the development of diabetic nephropathy. Other immune cells did not reveal significant changes in our study, and more research is needed to investigate the functions these immune cells play in DN.

Despite this, there are a number of drawbacks to our study. To begin, the sample size is not particularly huge; therefore, it is necessary to do extensive clinical tests. Second, the possible roles that DUSP1, PRDX6, and S100A8 may have in the development of DN were not examined in this research.

## Conclusion

Taken together, we first established a unique diagnostic model that was based on oxidative stress-relate genes for DN patients. This model offered provided a novel insight into the pathologic analyses and diagnostic biomarker exploration at the cellular and molecular levels.

## Data availability statement

The datasets presented in this study can be found in online repositories. The names of the repository/repositories and accession number(s) can be found in the article/supplementary material.

## Ethics statement

The studies involving human participants were reviewed and approved by South China Hospital. The patients/participants provided their written informed consent to participate in this study.

## Author contributions

H-MZ and NL analyzed the data and wrote the manuscript. D-XS helped data discussion. NL provided specialized expertise and collaboration in data analysis. H-MZ and LL conceived and designed the whole project and drafted the manuscript. All authors contributed to the article and approved the submitted version.
